# The Ross/Ross-Konno operation in neonates and infants: A salvage strategy and a durable repair

**DOI:** 10.1016/j.xjon.2025.03.010

**Published:** 2025-03-24

**Authors:** John M. Karamichalis, Morgan K. Moroi, Alice V. Vinogradsky, Edward Buratto, Priyanka Asrani, Diana Vargas Chaves, Andrew B. Goldstone, David Kalfa, Emile A. Bacha

**Affiliations:** a Section of Pediatric and Congenital Cardiac Surgery, Department of Surgery, New York Presbyterian-Morgan Stanley Children's Hospital, New York, NY; b Division of Cardiology, Department of Pediatrics, New York Presbyterian-Morgan Stanley Children's Hospital, New York, NY

**Keywords:** Ross operation, aortic valve disease, neonate, infant

## Abstract

**Objective:**

To review a single-center experience of the Ross operation in neonates and infants with severe aortic valve disease.

**Methods:**

Retrospective review identified patients younger than age 1 year who underwent Ross operation between 2010 and 2024. Primary outcome was cumulative incidence of death with transplant as a competing risk. Early and midterm outcomes were analyzed, including postoperative complications and reinterventions. A subgroup analysis of patients who remained hospitalized until Ross procedure was performed. Median follow-up was 5.7 years (interquartile range, 2.9-8.8. years).

**Results:**

Twenty-nine patients (5 neonates and 24 infants) underwent the Ross operation, 24 (82.8%) of whom had a Konno procedure. Median age was 3.5 months (interquartile range, 1.1-5.7 months). Median weight was 4.9 kg (interquartile range, 3.9-6.0 kg). Five patients (27.2%) were born with isolated critical aortic stenosis, whereas 24 patients had other complex left-sided lesions. Twenty-five patients (86.2%) had prior aortic or aortic valve procedures: 14 balloon valvuloplasty, 3 open valvotomy, 1 surgical valve repair, 8 interrupted arch repairs, 5 coarctation or arch repairs, and 2 subaortic stenosis repairs. A subset (n = 11) could not be discharged from the hospital, mostly due to residual valve disease after balloon dilation, and underwent salvage Ross. Nineteen patients (65.5%) had concomitant operations. There was 1 in-hospital and 1 late mortality. Two patients required transplant. At follow-up, 1 patient had moderate or greater neoaortic insufficiency requiring reintervention.

**Conclusions:**

The Ross operation can be performed in neonates and infants with excellent midterm outcomes. This operation can be safely offered as an exit strategy in neonates and infants with residual aortic valve disease who are unable to be discharged.

## Graphical Abstract

**Figure:**
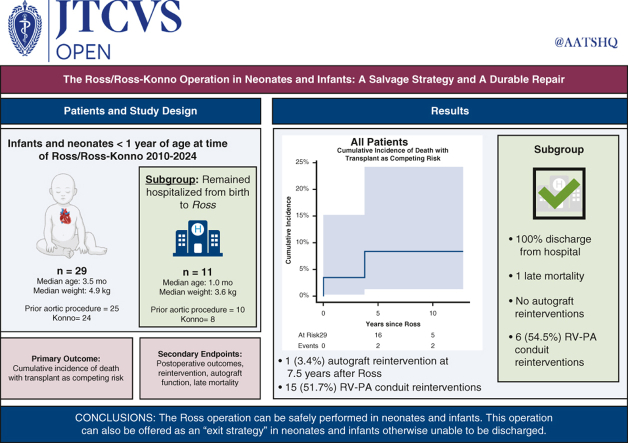

**Figure:**
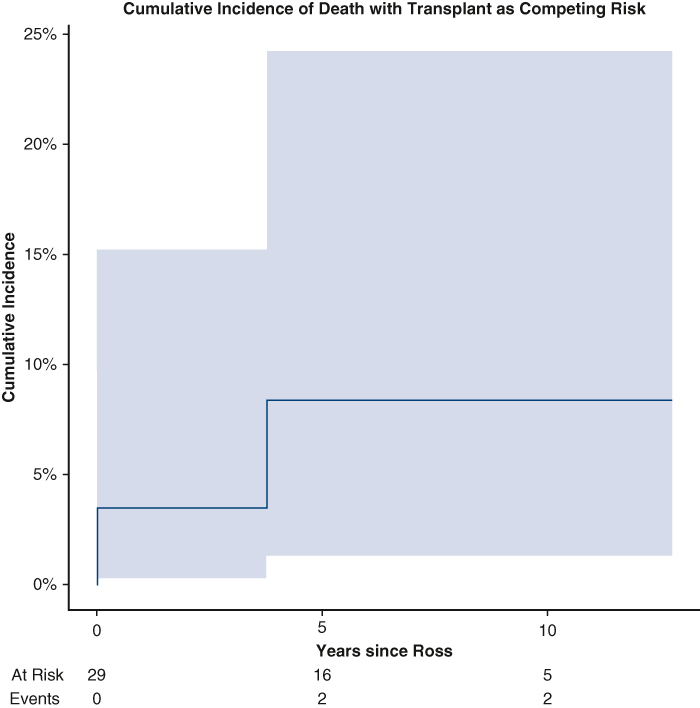
Cumulative incidence of death with transplant as a competing risk in infants following Ross.

**Central Message**

The Ross operation can be safely performed in neonates and infants with acceptable in-hospital mortality and excellent midterm outcomes.

**Perspective**

The Ross procedure can be performed in infants and neonates with acceptable in-hospital mortality and excellent midterm outcomes. For the subset of patients who cannot be discharged due to their critical aortic valve disease, the Ross operation may provide an exit strategy, allowing patients to be discharged from the hospital.

The management of congenital aortic stenosis in infants and neonates remains a challenge for congenital heart surgeons. Although transcatheter balloon dilation or surgical valvotomy may be considered as a first-choice treatment in neonates and infants with critical aortic stenosis, replacement of the aortic valve may become necessary with time. Unfortunately, there are limited options for aortic valve replacement in young pediatric patients. Aortic valve replacements utilizing porcine, homograft, or mechanical valve substitutes have been performed but shown to have poor outcomes. Biological prostheses and homografts undergo accelerated degeneration and structural failure,^1^, ^2^, ^3^, ^4^, ^5^ whereas long-term anticoagulation necessary for mechanical valves has been associated with increased thromboembolic and hemorrhagic complications.^1^^,^^2^ Further, all of these options fail to grow with the child.

Considering these limitations, the Ross procedure presents a favorable opportunity—an autologous valve substitute that allows for excellent hemodynamics, growth, and adaptive remodeling. First described in 1967 in adult patients,^6^ the Ross procedure has become a viable and robust option for aortic valve replacement in pediatric patients.^7^, ^8^, ^9^, ^10^, ^11^, ^12^, ^13^, ^14^, ^15^, ^16^ High operative mortality in infant and neonate patients, aortic insufficiency secondary to progressive dilation of the neoaortic root, and reoperation on the left ventricular outflow tract (LVOT) or right ventricular outflow tract remain ongoing concerns.^7^, ^8^, ^9^, ^10^, ^11^, ^12^, ^13^, ^14^, ^15^, ^16^ Herein, we describe our institutional experience with the Ross-Konno procedure in young infants and neonates with regard to early and midterm morbidity, mortality, and rates of reintervention.

## Methods

### Study Design

The Institutional Review Board of Columbia University Irving Medical Center approved this study (AAAR3478; approved August 12, 2022). A waiver of informed consent was obtained. A retrospective review of 29 patients younger than age 1 year who underwent the Ross operation at our institution between 2010 and 2024 was performed. A subgroup analysis of 11 patients who remained hospitalized after birth before undergoing Ross operation was conducted to assess the ability of the Ross procedure to act as an exit strategy for this population. Patients were only considered to be in the subgroup if they had care requirements necessitating hospitalization, such as poor LV function requiring inotropic support or respiratory failure requiring mechanical ventilation.

### Data Collection

Electronic medical records were reviewed to obtain detailed demographic, echocardiographic, operative, and clinical data, including in-hospital postoperative complications. Postdischarge data, including follow-up echocardiographic data, instances of reintervention on the pulmonary autograft or right ventricle to pulmonary artery (RV-PA) conduit, as well as survival status, were collected through review of the electronic medical record or by contacting patients and their primary cardiologists. Echocardiographic parameters were collected from diagnostic cardiology reports and measured according to the American Society of Echocardiography guidelines. Degrees of aortic stenosis and aortic insufficiency were graded at the level of the aortic valve for consistency. For each patient, echocardiographs were limited to 1 per distinct time period: 0 to 1 month, 3 to 6 months, 6 to 9 months, 9 to 12 months, 1 to 3 years, 3 to 5 years, 5 to 10 years, and 10 to 15 years. For patients with multiple echocardiographs per time period, the earliest complete echocardiogram results were selected for incorporation into the study. When applicable, aortic and pulmonary root dimensions were calculated using the Boston *z*-score method.^17^^,^^18^ For survivors to discharge, all patients had postdischarge echocardiographic data available. Follow-up was considered to be 88.9% complete in terms of patients who had echocardiographic data available from 2022 or later. Median follow-up was 5.7 years (interquartile range [IQR], 2.9-8.8 years).

### Study End Points

The primary outcome of interest was the cumulative incidence of death with transplant as competing risk. Secondary outcomes included early postoperative complications, reintervention on the autograft, reintervention on the RV-PA conduit, transplant, and late mortality. For patients who experienced a postoperative complication associated with increased neurodevelopmental risk (ie, stroke, cardiac arrest, or extracorporeal membrane oxygenation [ECMO]), late neurodevelopmental outcomes were examined.

### Patient Selection and Operative Technique

Our institutional preference for suitable valves with aortic stenosis is to proceed with percutaneous balloon valvuloplasty as an initial intervention, if the aortic annulus is of sufficient size. Similarly, for recurrent aortic valve stenosis, percutaneous balloon valvuloplasty may be reconsidered. In patients who may not be ideal candidates for balloon valvuloplasty or may have other concomitant lesions necessitating surgical intervention, open surgical valvuloplasty or open aortic valve repair (involving surgical valvuloplasty and other techniques such as aortic valve leaflet shaving) may be considered. However, if the obstruction is subaortic or the primary concern is significant regurgitation, then we elect to proceed with the Ross/Ross-Konno operation. During the study period, 5 surgeons performed the Ross procedure for this patient population.

Operative techniques for the Ross and Ross-Konno operations have been previously described.^19^^,^^20^ In short, the procedure is performed through a standard midline sternotomy, using standard techniques of cardiopulmonary bypass with bicaval or single venous cannulation with moderate systemic hypothermia. Antegrade cerebral perfusion is utilized in cases of arch reconstruction. The pulmonary root is harvest with either a beating heart or following crossclamp application and cardioplegia delivery. Once harvested, the pulmonary root is measured with a Hegar dilator to ensure there is no size mismatch with the native aortic root. We generally accept around 30% size mismatch. The autograft is trimmed and implanted at the subannular level using interrupted monofilament mattress sutures. The coronary arteries are implanted as buttons into their respective sinuses, and the distal anastomosis is performed in standard fashion. Next, an appropriately sized RV-PA conduit is sewn into the right ventricular outflow tract position. When re-establishing RV-PA continuity, our institutional preference is to use the largest possible size acceptable for the size and weight of the patient to maximize the potential lifespan of the conduit. When available in the appropriate size, we favor reconstruction with pulmonary homograft. However, when unavailable, we will use an aortic homograft or a Contegra (Medtronic) conduit.

When significant LVOT obstruction is present, a modified Konno incision is carried out to the left of the right coronary artery into the interventricular septum, with the length of the incision dependent on the nature of the LVOT obstruction. During implantation of the pulmonary autograft, the right ventricle muscle cuff is used to close the septal defect.

Mitral valve repair and endocardial fibroelastosis (EFE) resection are performed as needed, and the decision to perform mitral valvuloplasty is at the discretion of the surgeon. In general, our institution aims to repair the valve if moderate or greater regurgitation is present, and the valve anatomically appears amenable to repair. We also elect to perform extensive EFE resection when there is significant fibrous tissue present that is restricting LV function. During the postoperative period, the patient's blood pressure is tightly controlled, and we generally target normal age-specific blood pressure values.

### Statistical Analysis

Continuous variables were assessed for normality and are expressed as median (IQR). Categorical variables are presented as proportions. Differences between groups were assessed using the Fisher exact test for categorical variables or the Mann-Whitney *U* test for nonnormally distributed continuous variables. Cumulative incidence curves for death were estimated using a competing risks approach to account for transplant as a competing event. Specifically, we computed the nonparametric cumulative incidence function using the *cuminc* function in the R package *cmprsk*.^21^ The cumulative incidence function and 95% CI were subsequently visualized using a *ggplot*-based framework (via *ggcuminc* function), with risk tables added to display the number of patients at risk at designated time points. Time was measured in years from the patient's Ross procedure. Time from Ross/Ross-Konno to incidence of reintervention (on the pulmonary autograft or RV-PA conduit) were assessed using the Kaplan-Meier method. Patients were censored at the time of last known follow-up, death, or transplant. Kaplan-Meier curves were terminated when the number at risk fell below 10% of the sample. Baseline characteristics with missing variables are detailed in Table E1. Statistical analysis was performed using R statistical software, version 2023.06.2+561 (R Foundation for Statistical Computing).

## Results

### Patient Demographic and Preoperative Characteristics

A total of 29 patients (5 neonates and 24 infants) met inclusion criteria and underwent the Ross operation at younger than age 1 year. Individual patient details are listed in Table 1. Baseline characteristics for all patients are summarized in Table 2. Eighteen (62.1%) patients were boys. Median age at time of repair was 3.5 months (IQR, 1.1-5.7 months) and median weight was 4.9 kg (IQR, 3.9-6.0 kg). Of these patients, 5 (17.2%) patients were born prematurely and 7 (24.1%) had associated genetic diagnoses. A majority of patients (64.3%) had a bicuspid aortic valve. The primary diagnosis was isolated critical aortic stenosis in 5 patients. However, most patients (n = 24 [82.8%]) had other complex left-sided lesions contributing to multilevel outflow tract obstruction, including subaortic stenosis (75.9%), interrupted aortic arch (27.6%), arch hypoplasia (37.9%), and coarctation of the aorta (24.1%).

**Table 1:** Detailed patient characteristics

Patient	Age (mo)	Wt (kg)	Primary diagnosis	Prior interventions	Remained hospitalized before Ross	Reason for inability to discharge	AV path at Ross	Aortic annulus (mm)	*z-* score	Procedure	RVOT conduit
1	8.0	5.7	Critical AS, CoA, Shone's complex	- BVP - CoA repair - BD of CoA	No	NA	Mixed	8	−0.5	Ross-Konno	PH
2	1.1	4.2	IAA with severe LVOTO, VSD, EFE	IAA + VSD repair	No	NA	AS	4	−4.7	Ross-Konno	PH
3	0.2	2.5	Critical AS, sub-AS, VSD, AA hypoplasia, CoA	None	Yes	Critical AS + sub-AS requiring CPAP and PGE infusion	AS	3.5	−4.7	Ross-Konno + VSD closure + AA/CoA repair	AH
4	10.3	6.5	IAA with severe LVOTO, VSD	- IAA repair + PA band - PA band takedown + ASD/VSD repair	No	NA	AS	4	−5.67	Ross-Konno	AH
5	7.1	7.4	IAA with severe LVOTO, VSD, CoA	- IAA + VSD repair - BD of CoA - LVOT resection + surgical VP	No	NA	AI	–	–	Ross-Konno + AA repair	Hancock
6	2.7	3.6	Critical AS	BVP	Yes	Sepsis, UVC extravasation on DOL4 requiring exploratory laparotomy, BVP on DOL7 c/b severe AI, ventilator dependence with poor weight gain requiring NGT feeds	AI	7.2	0.05	Ross	PH
7	4.1	8.0	IAA with severe LVOTO, VSD	IAA + VSD repair	No	NA	AS	5	−5.12	Ross-Konno + AA repair	Contegra
8	4.4	6.0	Critical AS	BVP	No	NA	Mixed	–	–	Ross	AH
9	1.5	3.9	Critical AS, sub-AS, AA hypoplasia, EFE	- BVP - BVP	Yes	Severe AS, mild-moderate AI with severely depressed LV function requiring inotropic support	Mixed	6.4	−1.47	Ross-Konno + EFE resection	PH
10	1.7	4.0	Critical AS, sub-AS, AA hypoplasia, EFE, MR/MS	- Fetal BVP - BVP	No	NA	Mixed	5.6	−2.61	Ross-Konno + EFE resection + MV repair (PM splitting)	PH
11	1.8	3.7	Critical AS	BVP	Yes	Moderate-severe AI after BVP with poor LV function and prolonged intubation. Now extubated on inotropic support with poor weight gain requiring NGT feeds	AI	6.1	−1.65	Ross	PH
12	2.8	4.4	Critical AS, MS/MR	BVP	No	NA	Mixed	6.3	−1.8	Ross + MV repair (PM splitting, debridement of secondary chordae)	PH
13	1.0	3.0	Critical AS	BVP	Yes	Persistent severe AS + moderate AI after BVP c/b pericardial effusion requiring drainage, prolonged intubation, failure to thrive. Now extubated with poor weight gain requiring NGT feeds	Mixed	5.7	−0.55	Ross-Konno	AH
14	3.5	5.1	Critical AS, sub-AS, AA hypoplasia, CoA, MR	- CoA repair - BVP	No	NA	Mixed	5.9	–	Ross-Konno + MV repair (posterior commisuroplasty)	AH
15	0.5	4.2	Critical AS, sub-AS, VSD, AA hypoplasia, CoA, Shone's complex	None	No	NA	AS	5.1	−3.34	Ross-Konno + VSD closure + AA repair	PH
16	3.0	6.7	IAA + IVS with severe LVOTO, large ASD, Shone's complex	IAA repair + AV repair + MV plasty + fenestrated ASD closure	No	NA	AS	7	−2.08	Ross-Konno + redo MV repair (debridement of secondary chordae)	PH
17	0.8	1.4	Critical AS, AA hypoplasia	BVP	Yes	BVP c/b aortic dissection, persistent AS, poor LV function requiring inotropic support, ventilator dependence	AS	–	–	Ross + AA repair	PH
18	5.8	5.8	Critical AS, sub-AS	BVP	No	NA	AS	6.1	−2.99	Ross-Konno	PH
19	7.0	6.0	IAA with severe LVOTO, VSD	IAA + VSD repair	No	NA	AS	5.3	−3.98	Ross-Konno + AA repair + VSD repair	PH
20	5.1	6.1	Critical AS	BVP	No	NA	AI	8.5	−0.5	Ross-Konno	PH
21	0.5	3.3	Critical AS, sub-AS, AA hypoplasia, EFE	- Fetal BVP - Arch augmentation + surgical VP	Yes	Persistent severe AS c/b poor LV function requiring inotropic support, ventilator dependence	AS	4.6	−3.5	Ross-Konno + EFE resection	PH
22	6.0	4.9	IAA with severe LVOTO, VSD	IAA + VSD repair	Yes	Ex-26-wk infant with chronic respiratory insufficiency and persistent severe LVOTO	AS	5.5	−2.9	Ross-Konno + AA repair	PH
23	0.5	4.1	Critical AS, sub-AS, EFE	- BVP - BVP	Yes	Moderate residual AS and severe AI requiring inotropic support, ventilator dependence	Mixed	4.5	–	Ross-Konno + EFE resection	PH
24	11.0	6.3	DORV, VSD, AS, sub-AS, left AA with hypoplasia, CoA, TR, MR	- PA banding - PDA stent - CoA stent - Aortic uncrossing, arch repair, DKS, PA plasty, BDG, RMBTS	No	NA	AS	5.5	−3.7	DKS takedown + Ross-Konno + VSD repair + MV repair (commisuroplasty) + TV repair + BTS takedown	PH
25	3.5	5.0	AA hypoplasia with severe LVOTO, VSD	Arch + VSD repair, LVOT myomectomy	No	NA	AS	6	−2.64	Ross-Konno + AA repair	PH
26	3.7	5.7	Congenital AS/AI, sub-AS	None	No	NA	Mixed	7.7	−0.51	Ross-Konno	AH
27	0.9	3.6	Critical AS, sub-AS, AA hypoplasia, EFE, MR	- Fetal BVP - Atrial balloon septostomy + BVP - Bilateral PA flow restrictors	Yes	Residual severe AS c/b cardiogenic shock and multisystem organ dysfunction (ventilator dependence, AKI, liver dysfunction) requiring inotropic support	AS	5.7	−2.29	Ross-Konno + AA repair + EFE resection + MV repair (PM splitting) + LPA plasty	PH
28	5.7	5.2	Critical AS, sub-AS, AA hypoplasia, VSD	Norwood/Sano	No	NA	AS	5	−4.2	Ross-Konno + VSD repair + AA repair + PA plasty	PH
29	4.1	4.9	IAA with severe LVOTO, VSD, TR	- IAA + supraAS + VSD repair - Closure of residual VSD, LVOT chordae release, surgical VP, TV repair - AA ballooning	Yes	Prior surgery c/b delayed chest closure, residual severe LVOTO, prolonged mechanical ventilation. Now extubated but with increased work of breathing and poor weight gain requiring NGT feeds	AS	5	−3.66	Ross-Konno + septal myectomy + MV repair (debridement of secondary chordae)	PH

*AV*, Aortic valve; *RVOT*, right ventricular outflow tract; *AS*, aortic stenosis; *CoA*, coarctation; *BVP*, balloon valvuloplasty; *BD*, balloon dilation; *NA*, not available; *PH*, pulmonary homograft; *IAA*, interrupted aortic arch; *LVOTO*, left ventricular outflow tract obstruction; *VSD*, ventriculoseptal defect; *EFE*, endocardial fibroelastosis; *AA*, aortic arch; *CPAP*, continuous positive airway pressure; *PGE*, prostaglandin E; *AH*, aortic homograft; *PA*, pulmonary artery; *ASD*, atrial septal defect; *VP*, valvuloplasty; *UVC*, umbilical venous catheter; *DOL4*, day of life 4; *DOL7*, day of life 7; *c/b*, complicated by; *AI*, aortic insufficiency; *NGT*, nasogastric tube; *LV*, left ventricle; *MR*, mitral regurgitation; *MS*, mitral stenosis; *MV*, mitral valve; *PM*, papillary muscle; *IVS*, intact ventricular septum; *DORV*, double outlet right ventricle; *TR*, tricuspid regurgitation; *PDA*, patent ductus arteriosus; *DKS*, Damus-Kaye-Stansel; *BDG*, bi-directional Glenn; *RMBTS*, right modified Blalock-Taussig shunt; *TV*, tricuspid valve; *BTS*, Blalock-Taussig shunt; *AKI*, acute kidney injury; *LPA*, left pulmonary artery.

**Table 2:** Baseline characteristics for neonatal and infant patients undergoing Ross procedure

Variable	All patients (N = 29)	Remained hospitalized before Ross (n = 11)	Discharged before Ross (n = 18)	*P* value
Male sex	18 (62.1)	8 (72.7)	10 (55.6)	.60
Age at surgery (mo)	3.5 (1.1-5.7)	1.0 (0.6-2.3)	4.2 (3.1-6.7)	.003
Weight at surgery (kg)	4.9 (3.9-6.0)	3.6 (3.2-4.0)	5.7 (5.0-6.3)	<.001
Premature	5 (17.2)	4 (36.4)	1 (5.6)	.10
Genetic syndrome				.800
DiGeorge syndrome	2 (6.9)	1 (9.1)	1 (5.6)	
Turner syndrome	1 (3.4)	0 (0.0)	1 (5.6)	
Other	4 (13.8)	2 (18.2)	2 (11.1)	
None	22 (75.9)	8 (72.7)	14 (77.8)	
Preoperative mechanical ventilation	8 (27.6)	5 (45.5)	3 (16.7)	.21
Preoperative inotropic support	7 (24.1)	6 (54.5)	1 (5.6)	.01
Preoperative prostaglandin E1	3 (10.3)	2 (18.2)	1 (5.6)	.65
Prior aortic or aortic valve intervention	25 (86.2)	10 (90.9)	15 (83.3)	.99
Other complex left-sided lesions				
Coarctation	7 (24.1)	2 (18.2)	5 (27.8)	.89
Interrupted aortic arch	8 (27.6)	2 (18.2)	6 (33.3)	.65
Arch hypoplasia	11 (37.9)	4 (36.4)	7 (38.9)	1.00
Subaortic stenosis/membrane	22 (75.9)	7 (63.6)	15 (83.3)	.45
Shone complex	3 (10.3)	0 (0.0)	3 (16.7)	.42
Mitral stenosis	3 (10.3)	0 (0.0)	3 (16.7)	.42
Fibroelastosis	6 (20.7)	4 (36.4)	2 (11.1)	.25
Aortic valve morphology				.73
Tricuspid	4 (14.3)	1 (9.1)	3 (17.6)	
Bicuspid	18 (64.3)	7 (63.6)	11 (64.7)	
Unicuspid	6 (21.4)	3 (27.3)	3 (17.6)	
Degree of preoperative AS				.84
None/trivial	3 (10.7)	1 (9.1)	2 (11.8)	
Mild	2 (7.1)	1 (9.1)	1 (5.9)	
Moderate	3 (10.7)	2 (18.2)	1 (5.9)	
Moderate-severe	4 (14.3)	1 (9.1)	3 (17.6)	
Severe	16 (57.1)	6 (54.5)	10 (58.8)	
Degree of preoperative AI				1.00
None/trivial	11 (39.3)	4 (36.4)	7 (41.2)	
Mild	4 (14.3)	2 (18.2)	2 (11.8)	
Mild-moderate	2 (7.1)	1 (9.1)	1 (5.9)	
Moderate	3 (10.7)	1 (9.1)	2 (11.8)	
Moderate-severe	3 (10.7)	1 (9.1)	2 (11.8)	
Severe	5 (17.9)	2 (18.2)	3 (17.6)	
Native aortic annulus diameter (mm)	5.7 (5.0-6.3)	5.6 (4.7-6.0)	5.8 (5.1-6.5)	.43
Native aortic annulus *z*-score	−2.8 (−3.8 to −1.6)	−2.3 (−3.5 to −1.5)	−3.0 (−4.1 to −1.9)	.34
Native pulmonic annulus diameter (mm)	9.8 (8.8-11.2)	9.5 (9.0-10.8)	10.4 (8.7-11.8)	.43
Native pulmonic annulus *z*-score	−0.1 (−1.1 to 1.2)	−0.02 (−0.7 to 1.2)	−0.9 (−1.2 to 1.2)	.56

Values are presented as n (%) or median (interquartile range). *AS*, Aortic stenosis; *AI*, aortic insufficiency.

Twenty-five (86.2%) patients had a prior aortic or aortic valve intervention: 14 balloon valvuloplasty, 3 open valvotomy, 1 surgical aortic valve repair of a unicuspid valve involving open valvotomy and leaflet shaving, 8 interrupted aortic arch repairs, 5 coarctation or aortic arch repairs, and 2 subaortic stenosis repairs. Before undergoing the Ross procedure, 4 patients had repeat balloon valvuloplasty before undergoing the Ross procedure. Two patients were initiated down the single-ventricle pathway before proceeding with a biventricular repair involving the Ross procedure. One patient presented to an outside hospital during the early neonatal period with poor feeding, respiratory distress, and signs of cardiogenic shock. The patient was intubated, started on prostaglandin E1 (PGE) and inotropic support, and found to have significant LVOT narrowing as well as coarctation of the aorta with a large ventricular septal defect (VSD). The patient underwent a Norwood operation with Sano shunt placement and was transferred to our center. Rather than continuing down the single-ventricle pathway, a biventricular repair involving the Ross operation was performed. The second patient was born with double-outlet right ventricle, noncommitted inlet VSD, tunnel subaortic stenosis, and hypoplastic left-sided aortic arch with circumflex aorta that underwent pulmonary artery banding following by patent ductus arteriosus stenting and eventual aortic uncrossing, Damus-Kaye-Stansel procedure, arch repair, bidirectional Glenn, and left modified Blalock-Taussig shunt. The patient was inadvertently extubated and experienced respiratory cardiac arrest that was complicated by sternal dehiscence requiring multiple sternal revisions. After undergoing stage II, the patient continued to have poor respiratory failure and remained intubated with poor oxygenation. Therefore, the decision was made to pursue a biventricular repair with the Ross operation, which allowed the patient to be discharged.

Preoperatively, 8 (27.6%) patients required mechanical ventilation, 7 (24.1%) patients were supported with inotropes, and 3 (10.3%) patients were receiving PGE infusion. Median native aortic annulus *z*-score at time of surgery was −2.8 (IQR, −3.8 to −1.6), and median native pulmonary annulus *z*-score was −0.1 (IQR, −1.1 to 1.2).

### Intraoperative Characteristics and Early Outcomes

Intraoperative details and early outcomes are described in Table 3. Of the 29 patients, the Konno enlargement procedure was performed in 24 (82.8%) cases. Other concomitant procedures at the time of Ross operation included 10 (34.5%) aortic arch augmentations, 5 (17.2%) endocardial fibroelastosis resections, and 7 (24.1%) mitral valve repairs. RV-PA continuity was most frequently established with a pulmonary homograft (n = 21). Otherwise, an aortic homograft (n = 6), Contegra conduit (n = 1), or Hancock (Medtronic) conduit (n = 1) was used. Median RV-PA conduit size was 11 mm (IQR, 9.5-13.5 mm). Median cardiopulmonary bypass and aortic crossclamp times were 205 minutes (IQR, 172-245 minutes) and 132 minutes (IQR, 120-164 minutes), respectively. Eleven (37.9%) patients (4 of 5 neonates, 7 of 24 infants) left the operating room with an open chest.

**Table 3:** Operative characteristics, perioperative outcomes, and late postoperative outcomes for neonatal and infantile patients undergoing Ross procedure

Variable	All patients (n = 29)	Remained hospitalized before Ross (n = 11)	Discharged before Ross (n = 18)	*P* value
Operative characteristics				
CPB time (min)	205 (172-245)	207 (182-227)	203 (168-264)	.91
Crossclamp time (min)	132 (120-164)	143 (129-163)	123 (115-163)	.39
Ross-Konno procedure	24 (82.8)	8 (72.7)	16 (88.9)	.54
Concomitant procedure				
Concomitant aortic arch reconstruction	10 (34.5)	4 (36.4)	6 (33.3)	1.00
Concomitant septal myectomy	1 (3.4)	1 (9.1)	0 (0.0)	.69
Concomitant EFE resection	5 (17.2)	4 (36.4)	1 (5.6)	.10
Concomitant mitral valve repair	7 (24.1)	2 (18.2)	5 (27.8)	.89
Perioperative outcomes				
In-hospital mortality	1 (3.4)	0 (0.0)	1 (5.6)	1.00
In-hospital transplant	1 (3.4)	1 (9.1)	0 (0.0)	.80
Perioperative complications				
ECMO	5 (17.2)	0 (0.0)	5 (27.8)	.16
Cardiac arrest	5 (17.2)	0 (0.0)	5 (27.8)	.16
Prolonged mechanical ventilation	6 (20.7)	3 (27.3)	3 (16.7)	.83
Permanent pacemaker	1 (3.4)	1 (9.1)	0 (0.0)	.80
Stroke	3 (10.3)	0 (0.0)	3 (16.7)	.42
Sternal wound infection	1 (3.4)	1 (9.1)	0 (0.0)	.80
Branch PA stent	2 (6.9)	0 (0.0)	2 (11.1)	.70
Hospital length of stay (d)	28.0 (11.0-57.0)	34.0 (28.0-110.0)	13.5 (7.5-38.5)	.009
Late postoperative outcomes				
Late mortality	1 (3.4)	1 (9.1)	0 (0.0)	.80
Late transplant	1 (3.4)	0 (0.0)	1 (5.6)	1.00
Late surgical reintervention				
Autograft reintervention	1 (3.4)	0 (0.0)	1 (5.6)	1.00
RV-PA conduit reintervention	15 (51.7)	6 (54.5)	9 (50.0)	1.00
Branch PA stenting	2 (6.9)	0 (0.0)	2 (11.1)	.70
Mitral valve reintervention	3 (10.3)	2 (18.2)	1 (5.6)	.65
Permanent pacemaker	1 (3.4)	0 (0.0)	1 (5.6)	1.00
Duration of total follow-up time (y)	5.7 (2.9-8.8)	3.8 (2.2-6.7)	7.4 (4.7-10.0)	.11

Values are presented as n (%) or median (interquartile range). *CPB*, Cardiopulmonary bypass; *EFE*, endocardial fibroelastosis; *ECMO*, extracorporeal membrane oxygenation; *PA*, pulmonary artery; *RV-PA*, right ventricle to pulmonary artery.

During the early postoperative period, there was 1 (3.4%) in-hospital mortality, 5 (17.2%) patients who experienced cardiac arrest, and 5 (17.2%) patients who required ECMO. Two patients left the operating room on ECMO: 1 patient had a bradycardic event while closing the chest and 1 patient was cannulated in the setting of high PA pressures (75% systemic) and difficulty ventilating. A third patient was cannulated to ECMO on postoperative day 1 after experiencing increased pressor requirements and significantly reduced cardiac function. On postoperative day 1, another patient experienced cardiac arrest that ultimately resulted in the 1 in-hospital mortality. In addition, 2 more patients experienced cardiac arrest from bradycardia during endotracheal tube suctioning, and 2 patients had a cardiac arrest triggered by pulmonary hypertensive crises: 1 of whom was managed with 48 hours of ECMO support, the other was resuscitated and improved with inhaled nitric oxide. Lastly, 1 patient developed severe biventricular dysfunction following Ross-Konno operation on day of life 14 requiring 3 weeks of ECMO support. Although this patient initially recovered, the patient had a prolonged hospitalization and experienced bradycardic arrest on postoperative day 137 in the setting of acidosis and septic shock, resulting in a second ECMO insertion and eventual transplant on postoperative day 140.

Regarding other early postoperative complications, 3 (10.3%) patients had evidence of stroke, with 2 strokes occurring in the setting of ECMO use. Six (20.7%) patients experienced prolonged mechanical ventilation for more than 7 days. Two (6.9%) patients required branch pulmonary artery stenting for residual stenosis. Out of the 24 patients who underwent the Ross-Konno procedure, 1 patient had a small residual VSD and underwent VSD closure along with RV-PA conduit exchange at 3 months postoperatively. Additionally, 1 (3.4%) neonate who underwent Ross-Konno operation with simultaneous arch repair required early permanent pacemaker implantation postoperatively. Median hospital length of stay for the entire cohort was 28 days (IQR, 11-57 days).

### Late Outcomes

**Figure 1:**
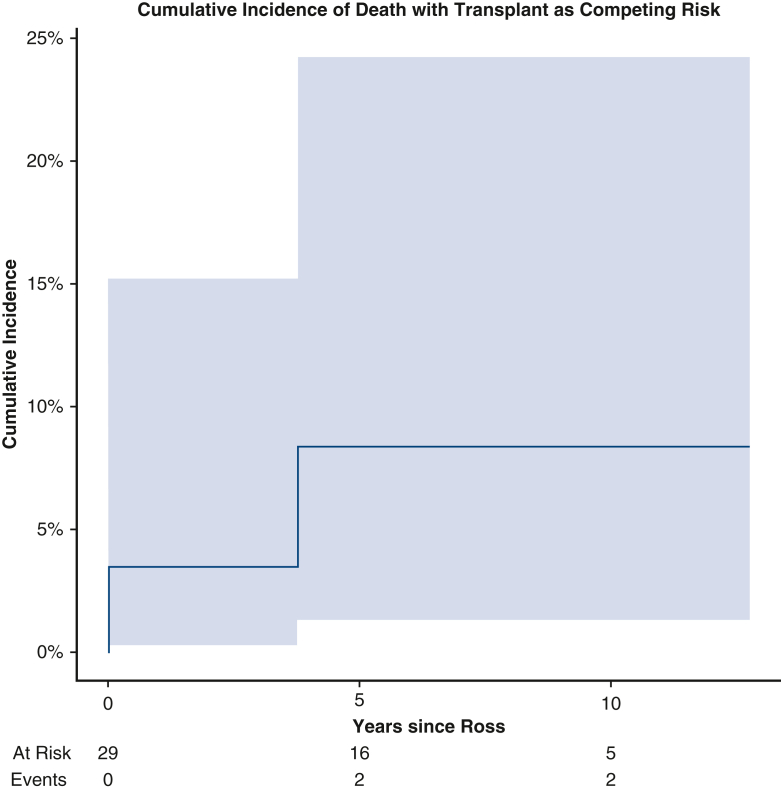
Cumulative incidence of death with transplant as competing risk. Numbers of patients at risk and number of events are included in the *lower* panel. *Shaded* 95% CIs are shown.

There was 1 late death reported 3.5 years postoperatively due to cardiac arrest on presentation to the emergency department in the setting of sepsis. One (3.4%) patient with a history of Shone's complex required late transplant at nearly 3 years postoperation. Following initial Ross-Konno operation with concomitant mitral valve repair, the patient required redo RV-PA conduit exchange and mitral valve replacement, which was complicated by complete heart block requiring permanent pacemaker implantation. The patient subsequently developed severe biventricular dysfunction necessitating biventricular assist device insertion and eventual transplantation. Before initiation of mechanical circulatory support, autograft performance remained in good standing with no stenosis and trivial regurgitation. Cumulative incidence of death with transplant as competing risk was 3.4% (95% CI, 0.2%-15.2%), 8.4% (95% CI, 1.3%-24.2%), and 8.4% (95% CI, 1.3%-24.2%) at 2, 4, and 8 years postoperatively, respectively (Figure 1).

**Figure 2:**
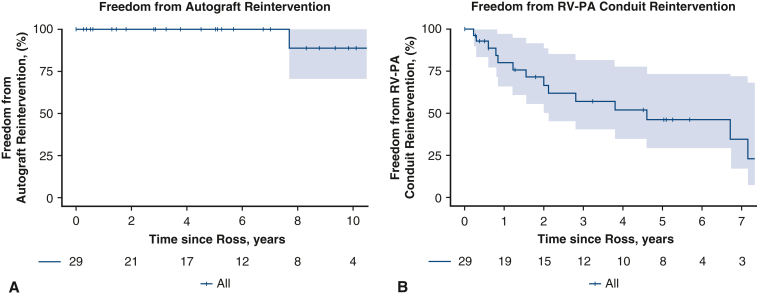
Freedom from reintervention on pulmonary autograft or right ventricle (A) to pulmonary artery (*RV-PA*) conduit (B) following Ross operation in all neonate and infant patients. Numbers of patients at risk are included in the *lower* panel. *Shaded* 95% CIs are shown.

Reintervention was not uncommon following the initial Ross/Ross-Konno operation, as shown in Table 3. As expected, most patients requiring reintervention had failing RV-PA conduits (n = 15 [51.7%]). Overall freedom from reintervention on the RV-PA conduit was 71.5% (95% CI, 55.6%-92.0%), 52.0% (95% CI, 34.8%-77.8%), and 46.3% (95% CI, 29.1%-73.6%) at 2, 4, and 6 years postoperatively, respectively (Figure 2, *B*). Autograft function was very well preserved. Longitudinal follow-up revealed only 1 patient who exhibited moderate or greater neoaortic insufficiency. The patient developed progressive and eventually severe neoaortic insufficiency without significant root dilation and ultimately underwent neoaortic valve replacement with the Ozaki technique 7.5 years after Ross operation. All other remaining autografts are performing well at latest follow-up with no significant stenosis and at most mild insufficiency. Overall freedom from reintervention on the autograft was 100.0%, 100.0%, and 88.9% (95% CI, 70.6%-100.0%) at 2, 4, and 8 years following Ross operation, respectively (Figure 2, *A*).

Other late reinterventions are outlined in Table 3. Three (10.3%) patients needed repeat mitral valve intervention, which resulted in 1 mechanical and 2 Melody (Medtronic) mitral valve replacements. One of these patients developed complete heart block requiring permanent pacemaker implantation. Two (6.9%) patients underwent branch PA stenting.

Given the high incidence of cardiac arrest, ECMO, and stroke in our cohort, midterm neurodevelopmental outcomes were examined in this specific cohort of patients following discharge from the hospital (n = 10). Patients who did not experience cardiac arrest, ECMO, or stroke were not assess for late neurodevelopmental outcomes. One patient who had an early spinal cord infarct after Ross-Konno procedure with aortic arch repair had mild residual motor defects at latest follow-up requiring a walker. One patient had an early stroke in the setting of ECMO after Ross-Konno procedure with aortic arch repair and VSD closure, and currently this patient has focal epilepsy and global developmental delay. One patient also had an early stroke in the setting of ECMO after the Ross-Konno procedure with aortic arch repair and VSD closure. At latest follow-up, the patient has mild global developmental delay. Lastly, 1 patient who underwent cardiac arrest and ECMO cannulation was noted to have mild developmental delay at latest follow-up. Six (60%) patients who experienced cardiac arrest, ECMO, and/or stroke did not exhibit any neurological deficits at latest follow-up.

### Subgroup Analysis: Patients Hospitalized From Birth With Residual Critical Aortic Valve Disease Until Ross/Ross-Konno

A total of 11 patients (72.7% boys) who remained hospitalized after birth underwent the Ross procedure before 1 year of life (Table 2). Four (36.4%) of these patients were premature and 3 patients (27.1%) were diagnosed with a genetic syndrome. Eight (72.7%) neonatal and infantile patients within the subgroup had other concomitant complex left-sided lesions. One patient with critical aortic stenosis directly underwent the Ross procedure on day of life 7. The remaining 10 (90.9%) patients had an aortic or aortic valve intervention before their Ross operation. All 10 of these patients had residual obstructive lesions postintervention that contributed to hindered clinical status and prevented discharge from the hospital (Table 1).

Before undergoing the Ross procedure, a larger proportion of patients who remained hospitalized from birth to Ross required mechanical ventilation (hospitalized: 5 [45.5%] vs discharged: 3 [16.7%]; *P* = .21), were supported with inotropes (hospitalized: 6 [54.5%] vs discharged: 1 [5.6%]; *P* = .01), and were receiving PGE infusion (hospitalized: 2 [18.2%] vs discharged: 1 [5.6%]; *P* = .65). Median age at time of surgery for patients who remained hospitalized was 1.0 months (IQR, 0.6-2.3 months), compared with 4.2 months (IQR, 3.1-6.7 months) in patients who were discharged before Ross (*P* = .003). Similarly, median weight at time of surgery was lower in patients hospitalized from birth to Ross procedure (hospitalized: 3.6 kg [IQR, 3.2-4.0 kg] vs discharged: 5.7 kg [IQR, 5.0-6.3 kg]; *P* < .001).

The predominate aortic valve pathology in both cohorts of patients was bicuspid with severe stenosis. Median native aortic annulus *z*-score at time of surgery was −2.3 (IQR, −3.5 to −1.5) and −3.0 (IQR, −4.1 to −1.9]) in the hospitalized and discharged cohorts, respectively (*P* = .34). Median native pulmonary annulus *z*-score was −0.02 (IQR, −0.7 to 1.2) and −0.9 (IQR, −1.2 to 1.2) in the hospitalized and discharged cohorts, respectively (*P* = .56). Operative details are displayed in Table 3. Most patients in both cohorts underwent the Konno procedure (72.7% vs 88.9%; *P* = .54). Similarly, a majority of patients underwent concomitant procedures (ie, aortic arch reconstructions [hospitalized: 36.4% vs discharged: 33.3%; *P* = 1.00], septal myectomy [9.1% vs 0.0%; *P* = .69], EFE resections [36.4% vs 5.6%; *P* = .10], and mitral valve repairs [18.2% vs 27.8%; *P* = .89]).

Subgroup analysis of the 11 patients who remained hospitalized after birth until Ross procedure showed that all survived to discharge home (0% in-hospital mortality), although 1 patient required cardiac transplantation for severe biventricular dysfunction following Ross operation and there was 1 late death due to cardiac arrest of unknown etiology, as previously described. Three (25.0%) patients experienced prolonged mechanical ventilation, 1 patient received a permanent pacemaker, and 1 patient developed a sternal wound infection. Median length of hospital stay was longer in patients hospitalized before Ross operation (hospitalized: 34.0 days [IQR, 28.0-110.0 days] vs discharged: 13.5 [IQR, 7.5-38.5 days]; *P* = .009).

There was 1 late death in the cohort hospitalized before Ross who presented to the hospital following cardiac arrest of unknown etiology. There was 1 late transplant in the cohort whose course following Ross was complicated by cardiac arrest requiring ECMO for suspected pulmonary hypertension crisis. This patient developed progressive mitral valve regurgitation and LV diastolic dysfunction 1.5 years after their Ross operation, prompting mechanical mitral valve replacement that was complicated by complete heart block and pacemaker placement. Eventually, the patient developed worsening biventricular failure requiring biventricular assist device insertion and underwent transplant 3 years after undergoing the Ross procedure. Overall cumulative incidence of death with transplant as a competing risk was 0.0%, 18.2% (95% CI, 0.4%-58.5%), and 18.2% (95% CI, 0.4%-58.5%) at 2, 4, and 6 years postoperatively, respectively.

**Figure 3:**
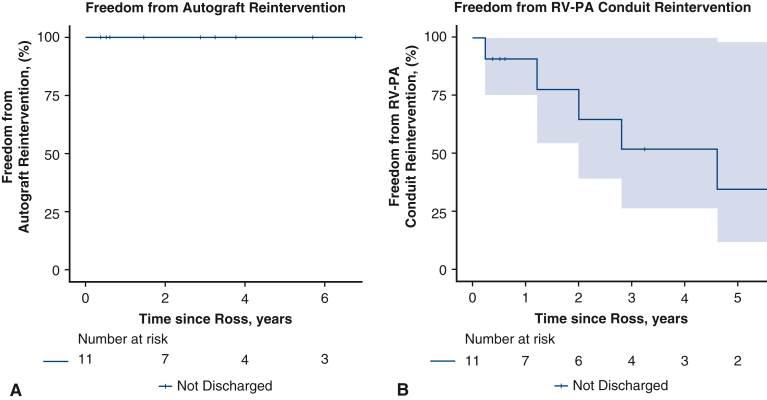
Freedom from reintervention on pulmonary autograft (A) or right ventricle to pulmonary artery (*RV-PA*) conduit (B) following Ross operation in all neonate and infant patients. Numbers of patients at risk are included in the *lower* panel. *Shaded* 95% CIs are shown.

No patients in the hospitalized subgroup required reintervention on the autograft (Figure 3, *A*), whereas 6 patients (54.5%) required reintervention on the RV-PA conduit. Overall freedom from reintervention on the RV-PA conduit was 77.9% (95% CI, 54.6%-100.0%) and 51.9% (95% CI, 26.6%-100.0%) at 2 and 4 years postoperatively, respectively (Figure 3, *B*). Other late complications included 2 patients (18.2%) who required reintervention on the mitral valve. Median follow-up duration for this cohort was 3.8 years (IQR, 2.2-6.7 years), compared with 7.4 years (IQR, 4.7-10.0 years) in the cohort discharged home before Ross operation.

## Discussion

The Ross procedure provides a promising surgical strategy for young infants and neonates with irreparable severe aortic valve disease or LVOT obstruction. Other current options provide significant size limitations and ongoing risk for complications. Mechanical aortic valve replacement increases risk of thrombotic events or bleeding from anticoagulation, whereas bioprosthetic valves and aortic homografts experience accelerated structural valve degeneration in pediatric populations, frequently necessitating early reintervention. Aortic valve replacement with the Ross procedure, or the patient's own pulmonary autograft, avoids thrombotic complications and the need for anticoagulation, as well as size limitations. By replacing the diseased valve with the patient's native tissue, the autograft can grow with the patient, mitigating the late complication of patient-prosthesis mismatch. The major potential disadvantage of this procedure is the act of placing both semilunar valves at risk for reintervention.

In neonates and infants, the pulmonary autograft has been shown to be a durable aortic valve substitute with excellent flow dynamics and the ability to match somatic growth.^4^^,^^5^^,^^9^, ^10^, ^11^, ^12^, ^13^, ^14^, ^15^, ^16^^,^^22^ Furthermore, autografts in children younger than age 1 year have been shown to undergo adaptive remodeling, allowing pulmonary autograft cells to differentiate and form functionally equivalent native aortic valve tissue.^23^

**Figure 4:**
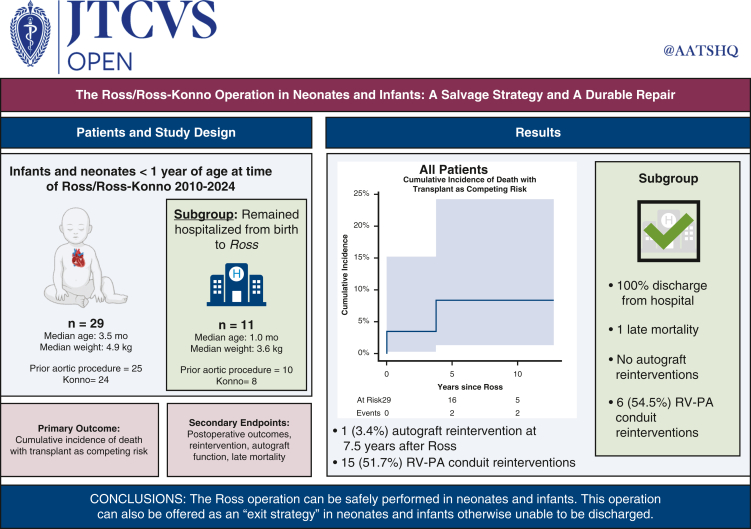
Summary of study findings. *RV-PA*, Right ventricle to pulmonary artery.

Herein, we report a series of 29 neonatal and infant patients who underwent the Ross procedure at younger than age 1 year with a median follow-up of 5.7 years (Figure 4). During the preoperative period, 86.2% of patients required an aortic or aortic valve intervention. A total of 20.7% of patients were mechanically ventilated, 13.8% of patients required inotropic support, and 10.3% were receiving continuous PGE infusion, showcasing the complex and critical condition of many neonates and infants with severe aortic valve disease and limited surgical options. Our results demonstrate that the Ross operation, along with other concomitant procedures, can be performed safely in these complex neonates and infants. The procedure carries significant risk of early morbidity within this vulnerable population; however, midterm results are favorable.

Overall survival in this cohort was 93.1%, with 1 early and 1 late death recorded. Despite excellent survival outcomes, significant postoperative support was required for these infants and neonates. Following Ross operation, 17.2% of patients required ECMO, 17.2% of patients experienced cardiac arrest, and 20.7% of patients experienced prolonged mechanical ventilation >7 days. Two patients required transplantation: 1 early and 1 late in the study period. Our results agree with published literature from other centers, demonstrating acceptable mortality rates but notable postoperative morbidity, and the risk for morbidity and prolonged hospitalization with Ross is important to keep in mind when counseling families and caretakers on the various options for aortic valve replacement.^4^^,^^9^, ^10^, ^11^, ^12^, ^13^, ^14^, ^15^, ^16^ On the other hand, it is important to recognize that, although there is appreciable early morbidity, many patients in our cohort did not exhibit late consequences of earlier sequelae (ie, autograft reintervention or neurodevelopmental delay), highlighting the long-term benefits of the Ross procedure in this population.

Many critics of the Ross procedure express concern about placing the 2 semilunar valves at risk for reintervention. However, 16 of 29 patients in our cohort had not yet required reintervention on the RV-PA conduit at time of latest follow-up. We recognize that many of these patients may be early in their postoperative course and that RV-PA conduit exchange is inevitable. However, the expected replacement of the RV-PA conduit was conducted in our cohort without added mortality. It is also worth noting that more and more of these interventions may become transcatheter as the arena of transcatheter intervention continues to advance, further decreasing the risks associated with these reinterventions.

More importantly, these results show appreciable functional autograft durability in neonates and infants with midterm follow-up. Most children exhibited none or only trivial neoaortic regurgitation at the time of latest follow-up, and only 1 patient (3.4%) required reintervention on the autograft for the duration of this study. Our series agrees with others, as few patients have required autograft reintervention, and most patients have demonstrated mild or less insufficiency at latest follow-up.^11^^,^^23^, ^24^, ^25^ Further, some studies have reported on the important consideration of autograft dilation over time. With a median follow-up of 6.4 years, our group previously demonstrated that autograft dilation occurs at the level of the aortic annulus and sinus in pediatric patients, including neonates and infants.^26^ More recently, in a 2024 multicenter center study of 133 neonates and infants with a longer median follow-up of 10.8 years, Greenberg and colleagues^27^ reported similar early dilatation of the autograft; however, this study showed that the autograft size normalized over time. Perhaps, it is the stabilization of the autograft over time that contributes to the remarkable freedom from autograft reintervention.

To assess the Ross procedure as an exit strategy for neonates and infants who remain hospitalized after birth, we performed a subgroup analysis of 11 infants and neonates within the study cohort. Almost all patients (90.9%) underwent an aortic or aortic valve intervention before the Ross procedure, and many of these patients required preoperative mechanical ventilation (45.5%), inotropic support (54.5%), and/or PGE (18.2%). Despite the challenges of surgery in this young, complex, and critically ill group of patients, all infants and neonates were able to be discharged home. One patient underwent successful cardiac transplantation and 2 patients required postoperative ECMO, but no deaths were recorded. Further, no patients in the subgroup required reintervention on the autograft during the study period. We believe that the Ross operation can be offered as an effective exit strategy with substantial hemodynamic advantages in select neonates and infants born with critical aortic valve disease who are otherwise unable to be discharged home and have very few alternative options. This observation is unique to our cohort study and has not been specifically demonstrated in other series.

Limitations of this study include those inherent to its retrospective design and single-center methodology. The sample size and number of outcome events precluded any univariable or multivariable analysis on predictors of death, transplant, or autograft reintervention. Echocardiographic data were not available for all patients at all time points. This, in combination with our limited sample size, hinders our ability in making meaningful conclusions related to autograft dimensions over time. Further, this study features many patients with outpatient cardiologists at other institutions, providing variations in surveillance and thresholds for reintervention. Lastly, late changes in autograft function have been shown beyond the reported observation period, and longer-term follow-up is warranted. It is crucial for us to continue to investigate the outcomes of autograft durability, the influence of the procedure on long-term survival, and risk factors for improving patient selection in this challenging population.

## Conclusions

The Ross procedure is an effective surgical repair strategy that can be performed successfully in infants and neonates with severe congenital aortic valve disease or LVOT obstruction. There remains a significant risk of early morbidity in this complex patient population; however, low mortality and transplant rates are achievable. Autograft durability remains excellent, and obligatory RV-PA conduit exchange can be performed safely in subsequent years. In patients with residual aortic valve disease who cannot be discharged, the Ross procedure may be a viable exit strategy.

## Conflict of Interest Statement

The authors reported no conflicts of interest.

The *Journal* policy requires editors and reviewers to disclose conflicts of interest and to decline handling or reviewing manuscripts for which they may have a conflict of interest. The editors and reviewers of this article have no conflicts of interest.

## Figures and Tables

**Table E1:** Baseline characteristics with missing variables

Variable	Missing
Aortic valve morphology	1
Degree of preoperative AS	1
Degree of preoperative AI	1
Native aortic annulus diameter (mm)	3
Native aortic annulus *z*-score	3
Native pulmonic annulus diameter (mm)	8
Native pulmonic annulus *z*-score	8

Values are presented as n. *AS*, Aortic stenosis; *AI*, aortic insufficiency.

## References

[1] Alsoufi B. (2014). Aortic valve replacement in children: options and outcomes. J Saudi Heart Assoc.

[2] Brown J.W., Ruzmetov M., Vijay P., Rodefeld M.D., Turrentine M.W. (2006). The Ross-Konno procedure in children: outcomes, autograft and allograft function, and reoperations. Ann Thorac Surg.

[3] Brown J.W., Ruzmetov M., Vijay P., Rodefeld M.D., Turrentine M.W. (2003). Surgery for aortic stenosis in children: a 40-year experience. Ann Thorac Surg.

[4] Maeda K., Rizal R.E., Lavrsen M. (2012). Midterm results of the modified Ross/Konno procedure in neonates and infants. Ann Thorac Surg.

[5] Williams I.A., Quaegebeur J.M., Hsu D.T. (2005). Ross procedure in infants and toddlers followed into childhood. Circulation.

[6] Ross D.N. (1967). Replacement of the aortic and mitral valves with a pulmonary autograft. Lancet.

[7] Rowe G., Gill G., Zubair M.M. (2023). Ross procedure in children: the Society of Thoracic Surgeons Congenital Heart Surgery Database Analysis. Ann Thorac Surg.

[8] Donald J.S., Wallace F.R.O., Naimo P.S. (2020). Ross operation in children: 23-year experience from a single institution. Ann Thorac Surg.

[9] Mookhoek A., Charitos E.I., Hazekamp M.G. (2015). Ross procedure in neonates and infants: a European multicenter experience. Ann Thorac Surg.

[10] Cleveland J.D., Bansal N., Wells W.J., Wiggins L.M., Kumar S.R., Starnes V.A. (2023). Ross procedure in neonates and infants: a valuable operation with defined limits. J Thorac Cardiovasc Surg.

[11] Luxford J.C., Ayer J.G., Betts K. (2022). The Ross/Ross-Konno procedure in infancy is a safe and durable solution for aortic stenosis. J Thorac Cardiovasc Surg.

[12] Sames-Dolzer E., Wickenhauser E., Kreuzer M. (2018). The Ross-Konno procedure in neonates and infants less than 3 months of age. Eur J Cardiothorac Surg.

[13] Rajab T.K., Zorrilla-Vaca A., Kavarana M.N., Mokashi S., Sainathan S. (2022). Ross operation in neonates: a meta-analysis. Ann Thorac Surg.

[14] Tohme S., Jiang S., Farooqi K. (2022). Ross procedure in neonate and infant populations: a meta-analysis review. World J Pediatr Congenit Heart Surg.

[15] Pasquali S.K., Cohen M.S., Shera D., Wernovsky G., Spray T.L., Marino B.S. (2007). The relationship between neo-aortic root dilation, insufficiency, and reintervention following the Ross procedure in infants, children, and young adults. J Am Coll Cardiol.

[16] Piccardo A., Ghez O., Gariboldi V. (2009). Ross and Ross-Konno procedures in infants, children and adolescents: a 13-year experience. J Heart Valve Dis.

[17] Sluysmans T., Colan S.D., Lai W.W., Cohen M.S., Geva T., Mertens L. (2009). Echocardiography in Pediatric and Congenital Heart Disease.

[18] Colan S.D., Lai W.W., Cohen M.S., Geva T., Mertens L. (2009). Echocardiography in Pediatric and Congenital Heart Disease.

[19] Mazine A., Ghoneim A., El-Hamamsy I. (2018). The Ross procedure: how I teach it. Ann Thorac Surg.

[20] Said S.M. (2021). The Ross-Konno procedure for congenital aortic stenosis. Ann Cardiothorac Surg.

[21] Gray R.J. (1988). A class of K-sample tests for comparing the cumulative incidence of a competing risk. Ann Stat.

[22] Elder R.W., Quaegebeur J.M., Bacha E.A., Chen J.M., Bourlon F., Williams I.A. (2013). Outcomes of the infant Ross procedure for congenital aortic stenosis followed into adolescence. J Thorac Cardiovasc Surg.

[23] Aikawa E., Aikawa M., Farber M. (2004). Clinical pulmonary autograft valves: pathologic evidence of adaptive remodeling in the aortic site. J Thorac Cardiovasc Surg.

[24] Alsoufi B., Al-Halees Z., Manlhiot C. (2010). Superior results following the Ross procedure in patients with congenital heart disease. J Heart Valve Dis.

[25] Hraska V., Krajci M., Haun Ch (2004). Ross and Ross-Konno procedure in children and adolescents: mid-term results. Eur J Cardiothorac Surg.

[26] Nguyen S.N., Bouhout I., Singh S. (2024). Long-term autograft dilation and durability after the Ross procedure are similar in infants, children, and adolescents with primary aortic stenosis. J Thorac Cardiovasc Surg.

[27] Greenberg J.W., Argo M., Ashfaq A. (2024). Long-term outcomes following the Ross procedure in neonates and infants: a multi-institutional analysis. J Thorac Cardiovasc Surg.

